# Impact of visceral obesity on infectious complications after resection for colorectal cancer: a retrospective cohort study

**DOI:** 10.1186/s12944-023-01890-4

**Published:** 2023-08-31

**Authors:** Wenshan Zhai, Yi Yang, Keyao Zhang, Lei Sun, Meng Luo, Xue Han, Min Wang, Zhiping Wang, Fang Gao

**Affiliations:** 1 Department of Anesthesiology, The Affiliated Hospital of Xuzhou Medical University, No.99 Huaihai West Road, Xuzhou, 221000 Jiangsu China; 2 Jiangsu Province Key Laboratory of Anesthesiology, Xuzhou Medical University, Tongshan, Xuzhou, 209 Jiangsu China; 3 Department of Anesthesiology, Suining Branch of Xuzhou Medical University Affiliated Hospital, No.2 Bayi West Road, Suining County, Xuzhou, Jiangsu China

**Keywords:** Visceral obesity, Colorectal cancer, Postoperative infectious complications, Predictive model, Machine learning

## Abstract

**Objectives:**

To explore the impact of visceral obesity (VO) measured by preoperative abdominal computed tomography (CT) on postoperative infectious complications for colorectal cancer (CRC) patients and establish a predictive model.

**Methods:**

Patients who underwent resection for colorectal cancer between January 2015 and January 2021 were enrolled in this study. All patients were measured for body mass index (BMI) and visceral fat area (VFA) preoperatively. Infectious complications were compared between the different groups according to BMI and VO categories. Univariate and multivariate logistic regression were used to analyze whether VO was an independent risk factor for postoperative infectious complications. According to the results of logistic regression, six machine learning approaches were used to establish predictive models and perform internal validation. The best-performing model was interpreted by the SHAPley Additive exPlanations value.

**Results:**

Approximately 64.81% of 520 patients had VO. VO was significantly connected with postoperative infectious complications (*P* < 0.001), coronary heart disease (*P* = 0.004), cerebral infarction (*P* = 0.001), hypertension (*P* < 0.001), diabetes (*P* < 0.001), and fatty liver (*P* < 0.001). The rates of wound infection (*P* = 0.048), abdominal or pelvic infection (*P* = 0.006), and pneumonia (*P* = 0.008) increased obviously in patients with VO. Compared to the low BMI group, a high BMI was found to be significantly associated with hypertension (*P*=0.007), fatty liver (*P*＜0.001), and a higher rate of postoperative infection (*P*=0.003). The results of logistic regression revealed that VO (OR = 2.01, 95% CI 1.17 ~ 3.48, *P* = 0.012), operation time ≥ 4 h (OR = 2.52, 95% CI 1.60 ~ 3.97, *P* < 0.001), smoking (OR = 2.04, 95% CI 1.16 ~ 3.59, *P* = 0.014), ostomy (OR = 1.65, 95% CI 1.04 ~ 2.61, *P* = 0.033), and chronic obstructive pulmonary disease (COPD) (OR = 2.23, 95% CI 1.09 ~ 4.57, *P* = 0.029) were independent risk factors. The light gradient boosting machine (LGBM) model displayed the largest area under the receiver operating characteristic curve (AUC) (0.74, 95% CI 0.68 ~ 0.81).

**Conclusions:**

In this study, VO was superior to BMI in evaluating the influence of obesity on metabolic comorbidities and postoperative infectious complications in colorectal cancer patients.

**Supplementary Information:**

The online version contains supplementary material available at 10.1186/s12944-023-01890-4.

## Introduction

Colorectal cancer (CRC), with over 1.8 million cases and 915,880 deaths annually, is the third most common cancer in the world [1]. Surgery is the main curative option, but up to 26% of patients experience postoperative infectious complications (PICs) [2], which can increase postoperative morbidity and mortality and result in longer hospital stays and higher medical expenses [3]. According to one meta-analysis about the types and severity of complications and long-term outcomes for colorectal cancer patients, postoperative infectious complications could serve as a predictor of outcomes and might recognize potential points of intervention and remediation to systematically improve postoperative outcomes [4].

Obesity is a well-known risk factor for postoperative infectious complications. According to a review published in the journal Lancet Infect Dis, patients with obesity were more susceptible to various infections, including postoperative infection [5]. Internationally, BMI is often used to measure obesity [6]. Almasaudi used BMI < 25 vs. ≥ 25 kg/m^2^ to explore the relationship between obesity and postoperative infectious complications for colorectal cancer patients from Asian countries, which demonstrated that obesity increased postoperative infectious complications by approximately 60% [7]. However, BMI cannot accurately assess the distribution of fat tissue and varies widely among individuals [8]. Hence, the relevance between BMI and postoperative infectious risk for colorectal cancer patients has recently been questioned, and the research focus has turned to visceral fat tissue accumulation.

Visceral adipose tissue (VAT), which is primarily distributed around the heart and intra-abdominal organs [9], produces more adipokines to induce a chronic inflammatory status and affects metabolism, inflammation, and vessels, and is the main source of low-grade systemic inflammation [10]. According to a clinical study on body composition and surgical outcomes for gastric cancer, VAT was associated with a higher postoperative level of serum C-reactive protein (CRP) and more postoperative complications [11]. As a result, it was recommended to use VO defined by visceral fat area (VFA) to assess the connection between obesity and postoperative infection internationally [12]. However, prior research has not reached a consensus about whether VO is a more appropriate indicator of obesity than BMI or the impact of VO on postoperative infection.

In our study, we aimed to identify the relationship between VO and metabolic comorbidities and postoperative infectious complications in colorectal cancer patients. Six machine learning approaches were applied to establish predictive models, which provided preventive guidance for clinical work.

## Patients and methods

### Subjects

We retrospectively analyzed 520 patients who underwent elective resection for colorectal cancer at the Affiliated Hospital of Xuzhou Medical University from January 2015 to January 2021. All patients underwent preoperative abdominal CT to exclude metastatic disease. This retrospective study was approved by the Ethical Review Board of the Affiliated Hospital of Xuzhou Medical University (XYFY2022 - KL043–01). Given that there was little to no danger to the participants in the study, informed consent was not needed. The waiver had no impact on participants’ rights and welfare. This project was registered in the China Clinical Trial Registry (NO. ChiCTR2200056470). Preoperative mechanical and antibiotic bowel preparation was performed. All patients were given prophylactic intravenous antibiotics.

The following were the criteria for inclusion: (1) pathological diagnosis of colorectal cancer; (2) age ≥ 18 years; (3) planned elective resection for colorectal cancer; and (4) available abdominal CT scans obtained in our hospital within 15 days before surgery. The exclusion criteria included (1) any CRC surgery in the emergent setting; (2) systemic inflammatory response syndrome before surgery; (3) intestinal obstruction; and (4) incomplete medical records. Flow diagram for the study selection process (Additional file. 1).

### Fat measurement

**Fig. 1:**
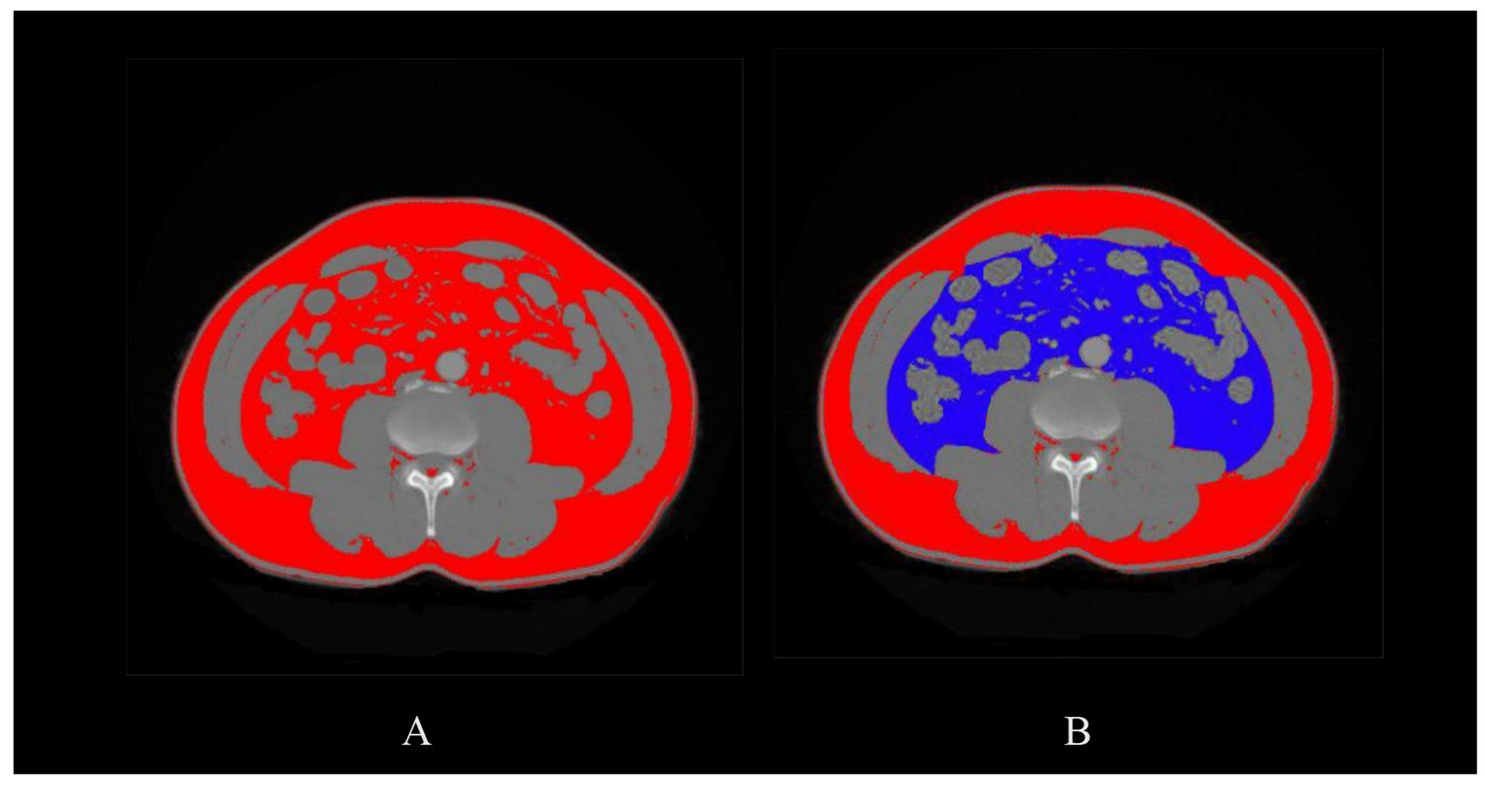
Measurement of the abdominal visceral fat area. **A** shows the total fat area (red area). **B** shows the different distributions of abdominal fat tissue; the blue area shows the visceral fat tissue, and the red area shows the subcutaneous fat tissue

Preoperative abdominal CT was performed in all patients. VFA and subcutaneous fat area (SFA) were measured at the level of L3~L4 by using CT images. Adipose tissue was determined by adjusting the attenuation level within the range of -190 to -30 Hounsfield units (HU) [13]. ImageJ [14] software was used to analyze the CT images and calculate the fat parameters (Fig. 1). According to the recommended VFA cutoff determined by the Japan Society for the Study of Obesity [15], VFA ≥ 100cm^2^ was regarded as VO. According to the World Health Organization (WHO), people with BMI ≥ 30 kg/m^2^ are considered obese. However, considering different body shapes and fat distributions in different ethnic populations, we adopted the Asian-Pacific standard: patients were divided into a high BMI group and a low BMI group at the cutoff value of 25 kg/m^[2 [16]]^.

### Definition of colorectal surgery-associated infection

Postoperative infectious complications were defined as any infection occurring during patients’ hospital stays, including surgical site infection (SSI) and non-SSI [17]. SSI meant incision and organ/space infection; the former indicated that the infection was specific to the wound, while the latter suggested that the infection was present in the surgical region (e.g., abdominal or pelvic infection and anastomotic fistula). Non-SSI included pneumonia and urinary tract infection.

### Patient characteristics and outcome variables

We retrospectively collected the following data: demographic characteristics (e.g., age, sex, BMI, and smoking), fat parameters (e.g., VFA and SFA), comorbidities (e.g., diabetes, cerebral infarction, COPD, hypertension, coronary heart disease, and fatty liver), preoperative biochemistry data (e.g., albumin, white blood cell count, neutrophil count, mononuclear cell count, lymphocyte count, preoperative fasting blood glucose, and hemoglobin (Hb)), pathological characteristics of tumors (e.g., maximum diameter, location, degree of differentiation, pathology, and stage), perioperative conditions (e.g., perioperative blood transfusion, operation time, surgical approach, ostomy) and postoperative outcomes (e.g., wound infection, pneumonia, urinary tract infection, abdominal or pelvic infection, anastomotic fistula, postoperative hospital stay and the rate of transfer to the intensive care unit after surgery).

### Statistical analysis

We utilized Python (version 3.9.7) for all statistical analyses. Anaconda software (version 2021.11, Anaconda Inc., Austin, Texas, USA) was employed to execute Python code. Continuous data are reported as medians with interquartile ranges, while categorical variables are expressed as numbers and percentages. Differences between groups were analyzed by the Mann-Whitney U test for continuous variables and the chi-squared test or Fisher’s exact test for categorical data. Univariate analysis identified correlations between postoperative infectious complications and potential risk factors (*P* < 0.05). Multivariate logistic regression analysis was utilized to identify independent risk factors for postoperative infectious complications. Significant features from univariate analysis were checked for multicollinearity before being included in the multivariate analysis by calculating the Spearman correlation coefficient [18]. *P* < 0.05 was considered statistically significant.

### Model establishment and evaluation

The data were divided randomly: 70% was used for training and 30% for validation. We utilized the following machine learning approaches to establish models, which were the most practical and popular for classification: gradient boosting decision tree (GBDT), extreme gradient boosting (XGBoost), decision tree (DT), random forest (RF), logistic regression (LR), and light gradient boosting machine (LGBM).

The GBDT algorithm consists of several decision trees, and the outcome is calculated by adding conclusions from all trees. DT is a tree-like structure model. Each internal node represents an attribute judgment, each branch represents the output of a judgment result, and each leaf node represents a classification result. XGBoost is considered an enhanced version of the GBDT algorithm and can be applied to the majority of regression and classification problems. RF is an ensemble classifier that combines several decision trees using majority voting. LR is a supervised machine learning technique that is mostly used to solve binary problems. LGBM is a framework to implement the GBDT algorithm, which has faster training speed, lower memory consumption, and improved accuracy.

To improve the accuracy of the models, we attempted a fivefold cross-validation grid search (GridSearchCV) [19] to exhaustively search specified parameter values for each estimator. The receiver operating characteristic (ROC) curve was recorded in each training model. Finally, the best-performing machine learning model was determined by comparing AUCs.

### Model interpretation

SHAPley Additive exPlanations (SHAP) values [20] were used to interpret the best-performing predictive model. SHAP values represented the contribution of features to predict infection. Red shows higher feature values, whereas lower feature values are represented in blue. When the SHAP value > 0, the feature has a positive effect on infection. In contrast, this feature has a negative effect. Features were ranked according to the average absolute SHAP values.

## Results

### Patient characteristics

The median age was 64.00 years, and 40.96% of patients were male. The mean BMI was 23.72 kg/m^2^, 30.38% were in the high BMI group, and 64.81% of patients had visceral obesity. Table 1 contains the demographic and clinical data of the enrolled participants. Patients with VO had more coronary heart disease (*P* = 0.004), cerebral infarction (*P* = 0.001), hypertension (*P* < 0.001), diabetes (*P* < 0.001), and fatty liver (*P* < 0.001). Compared with the low BMI group, the rates of hypertension and fatty liver were more often higher in the high BMI group.

**Table 1:** Basic and clinical data in the general population and subgroups according to VO and BMI categories

	Total study population (N = 520)	Low BMI (N = 362)	High BMI (N = 158)	*P* value	Non-VO (N = 183)	VO (N = 337)	*P* value
Age ≥ 65 years	256 (49.23)	184 (50.83)	72 (45.57)	0.270	72 (39.34)	184 (54.60)	0.001^*^
Gender (male)	213 (40.96)	150 (41.44)	63 (39.87)	0.739	88 (48.09)	125 (37.09)	0.015^*^
Smoking	80 (15.38)	59 (16.30)	21 (13.29)	0.382	22 (12.02)	58 (17.21)	0.117
Hypertension	164 (31.54)	101 (27.90)	63 (39.87)	0.007^*^	27 (14.75)	137 (40.65)	0.000^*^
Diabetes	65 (12.50)	39 (10.77)	26 (16.46)	0.072	9 (4.92)	56 (16.62)	0.000^*^
COPD	44 (8.46)	36 (9.94)	8 (5.06)	0.066	19 (10.38)	25 (7.42)	0.246
Coronary heart disease	56 (10.77)	37 (10.22)	19 (12.03)	0.542	10 (5.46)	46 (13.65)	0.004^*^
Cerebral infarction	73 (14.04)	51 (14.09)	22 (13.92)	0.960	13 (7.10)	60 (17.80)	0.001^*^
Fatty liver	27 (5.19)	10 (2.76)	17 (10.76)	0.000^*^	1 (0.55)	26 (7.72)	0.000^*^
Anemia	158 (30.38)	119 (32.87)	39 (24.68)	0.062	59 (32.24)	99 (29.38)	0.498
Low albumin	173 (33.27)	131 (36.19)	42 (26.58)	0.033^*^	67 (36.61)	106 (31.45)	0.233
Fasting blood glucose	5.31 (4.88, 5.81)	5.31 (4.80, 5.68)	5.38 (5.06,6.13)	0.000^*^	5.20 (4.71, 5.46)	5.31 (4.98, 6.04)	0.000^*^
WBC (×10^9^ /L)	6.20 (5.10, 7.30)	6.00 (5.10, 7.10)	6.40 (5.30, 7.50)	0.110	5.90 (4.90, 7.05)	6.30 (5.20, 7.50)	0.077
Neutrophil (×10^9^ /L)	3.78 (2.91, 4.76)	3.71 (2.92, 4.67)	3.86 (2.89, 4.92)	0.397	3.66 (2.94, 4.55)	3.85 (2.90, 4.85)	0.472
LMR	4.58 (3.48, 5.94)	4.41 (3.23, 5.90)	4.80 (3.85, 6.05)	0.009^*^	4.36 (3.30, 5.92)	4.63 (3.53, 5.94)	0.349
Operation time ≥ 4 h	174 (33.46)	108 (29.83)	66 (41.77)	0.008^*^	55 (30.05)	119 (35.31)	0.225
Surgical approach				0.687			0.146
Laparoscopy	463 (89.04)	321 (88.67)	142 (89.87)		158 (86.34)	305 (90.50)	
Open	57 (10.96)	41 (11.33)	16 (10.13)		25 (13.66)	32 (9.50)	
Perioperative blood infusion	76 (14.62)	57 (15.75)	19 (12.03)	0.269	29 (15.85)	47 (13.95)	0.558
Ostomy	165 (31.73)	109 (30.11)	56 (35.44)	0.230	55 (30.05)	110 (32.64)	0.545
Pathology				0.485			0.205
Adenocarcinoma	443 (85.19)	311 (85.91)	132 (83.54)		151 (82.51)	292 (86.65)	
Nonadenocarcinoma	77 (14.81)	51 (14.01)	26 (16.46)		32 (17.49)	45 (13.35)	
Tumor location				0.811			0.416
Rectum	246 (47.31)	170 (46.96)	76 (48.10)		91 (49.73)	155 (45.99)	
Colon	274 (52.69)	192 (53.04)	82 (51.90)		92 (50.27)	182 (54.01)	
Histologic type				0.006^*^			0.643
Low	119 (22.88)	95 (26.24)	24 (15.19)		44 (24.04)	75 (22.26)	
Medium-high	401 (77.12)	267 (73.76)	134 (84.81)		139 (75.96)	262 (77.74)	
Tumor diameter (Max)(cm)	4.50 (3.50, 6.00)	4.50 (3.65, 6.00)	4.50 (3.50, 5.90)	0.305	4.80 (4.00, 6.00)	4.50 (3.50, 5.60)	0.054
Pathological stage				0.398			0.005^*^
< II B stage	216 (41.54)	146 (40.33)	70 (44.30)		61 (33.33)	155 (45.99)	
≥ II B stage	304 (58.46)	216 (59.67)	88 (55.70)		122 (66.67)	182 (54.01)	

Anemia, hemoglobin concentration < 120 g/L for men and < 110 g/L for women

Low albumin, albumin < 40 g/L; BMI, body mass index; VO, visceral obesity, visceral fat area ≥ 100 cm^2^; High BMI, BMI ≥ 25 kg/m^2^; SFA, subcutaneous fat area; COPD, chronic obstructive pulmonary disease; LMR, lymphocyte to monocyte ratio

Data are presented as numbers (%) or medians (interquartile range)

^*^Compared with the low BMI group, *P* < 0.05

^*^Compared with the non-VO group, *P* < 0.05

**Fig. 2:**
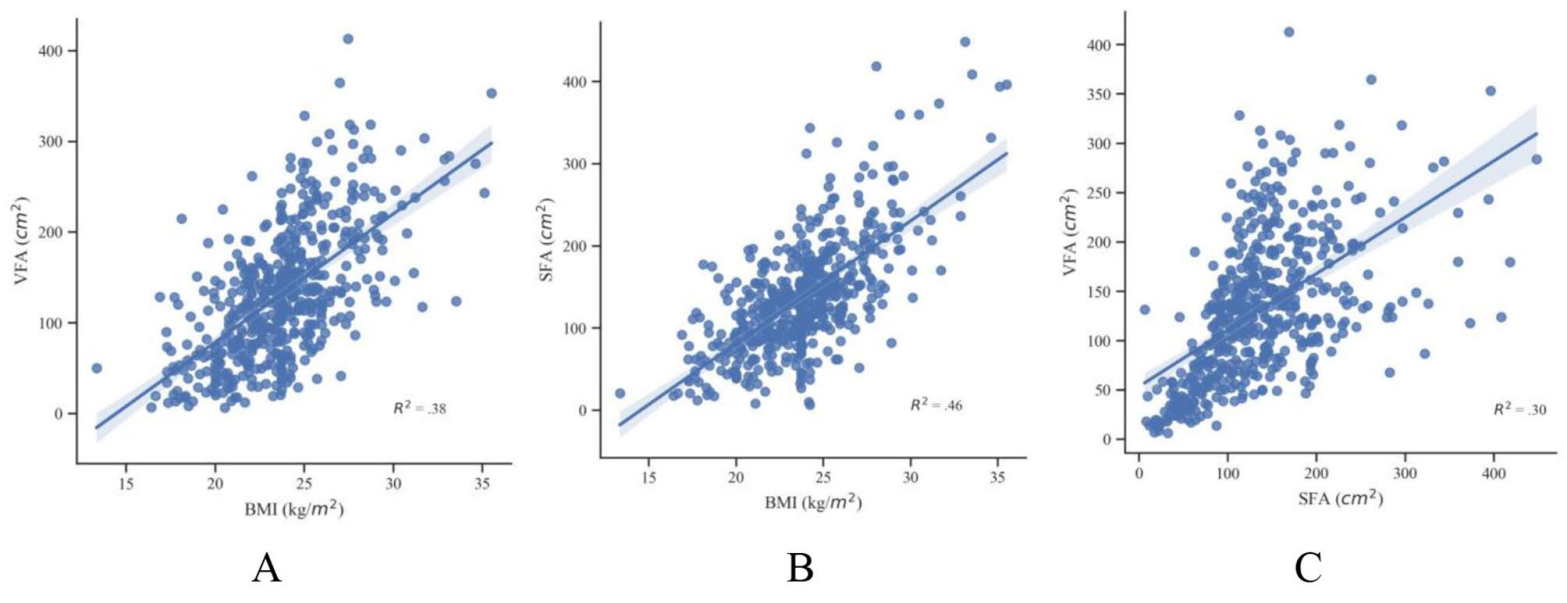
Correlations between BMI, VFA, and SFA. BMI, body mass index; VFA, visceral fat area; SFA, subcutaneous fat area

Figure 2 shows the relationship among fat parameters. Linear regression analysis demonstrated a positive correlation between BMI and VFA (R^2^ = 0.38, *P* < 0.01) (Fig. 2A), BMI and SFA (R^2^ = 0.46, *P* < 0.01) (Fig. 2B), as well as SFA and VFA (R^2^ = 0.30, *P* < 0.01) (Fig. 2 C).

### Short-term postoperative outcomes

Postoperative total infectious complications occurred in 23.27% of the patients. Table 2 displays the postoperative results according to BMI and VO categories. Patients with VO (*P* < 0.001) or high BMI (*P* = 0.003) had a higher incidence of total infectious complications than their opposite groups. Patients with VO had more wound infections (*P* = 0.048), abdominal or pelvic infections (*P* = 0.006), and pneumonia (*P* = 0.008). Considering BMI categories, no differences in anastomotic leakage, wound infection, abdominal or pelvic infection, and pneumonia were observed. Moreover, no differences were detected for postoperative hospital stays and the rate of transfer to the ICU after surgery when patients were stratified according to BMI or VO.

**Table 2:** Postoperative outcomes in the general population and subgroups according to BMI and VO categories

	Total study population (N = 520)	Low BMI (N = 362)	High BMI (N = 158)	*P* value	Non-VO (N = 183)	VO (N = 337)	*P* value
Infectious complications	121(23.27)	71(19.61)	50(31.65)	0.003^*^	25(13.66)	96(28.49)	0.000^*^
Anastomotic leakage	20(3.85)	13(3.59)	7(4.43)	0.647	9(4.92)	11(3.26)	0.349
Wound infection	28(5.38)	16(4.42)	12(7.59)	0.140	5(2.73)	23(6.82)	0.048^*^
Abdominal or pelvic infection	23(4.42)	13(3.59)	10(6.33)	0.163	2(1.09)	21(6.23)	0.006^*^
Pneumonia	46(8.85)	27(7.46)	19(12.03)	0.092	8(4.37)	38(11.28)	0.008^*^
Urinary infection	4(0.77)	2(0.55)	2(1.27)	0.392	1(0.55)	3(0.89)	0.668
Postoperative hospital stays (d)	13.00(11.00, 16.00)	13.00(11.00, 16.00)	12.00(11.00, 15.00)	0.720	13.00(11.00,15.00)	13.00(11.00, 16.00)	0.878
Post-operative ICU	49(9.42)	36(9.94)	13(8.23)	0.380	12(6.56)	37(10.98)	0.099

VO, visceral obesity, visceral fat area ≥ 100 cm^2^; High BMI, BMI ≥ 25 kg/m^2^.

Data are presented as numbers (%) or medians (interquartile range)

^*^Compared with the low BMI group, *P* < 0.05

^*^Compared with the non-VO group, *P* < 0.05

### Risk factors for postoperative infection

**Fig. 3:**
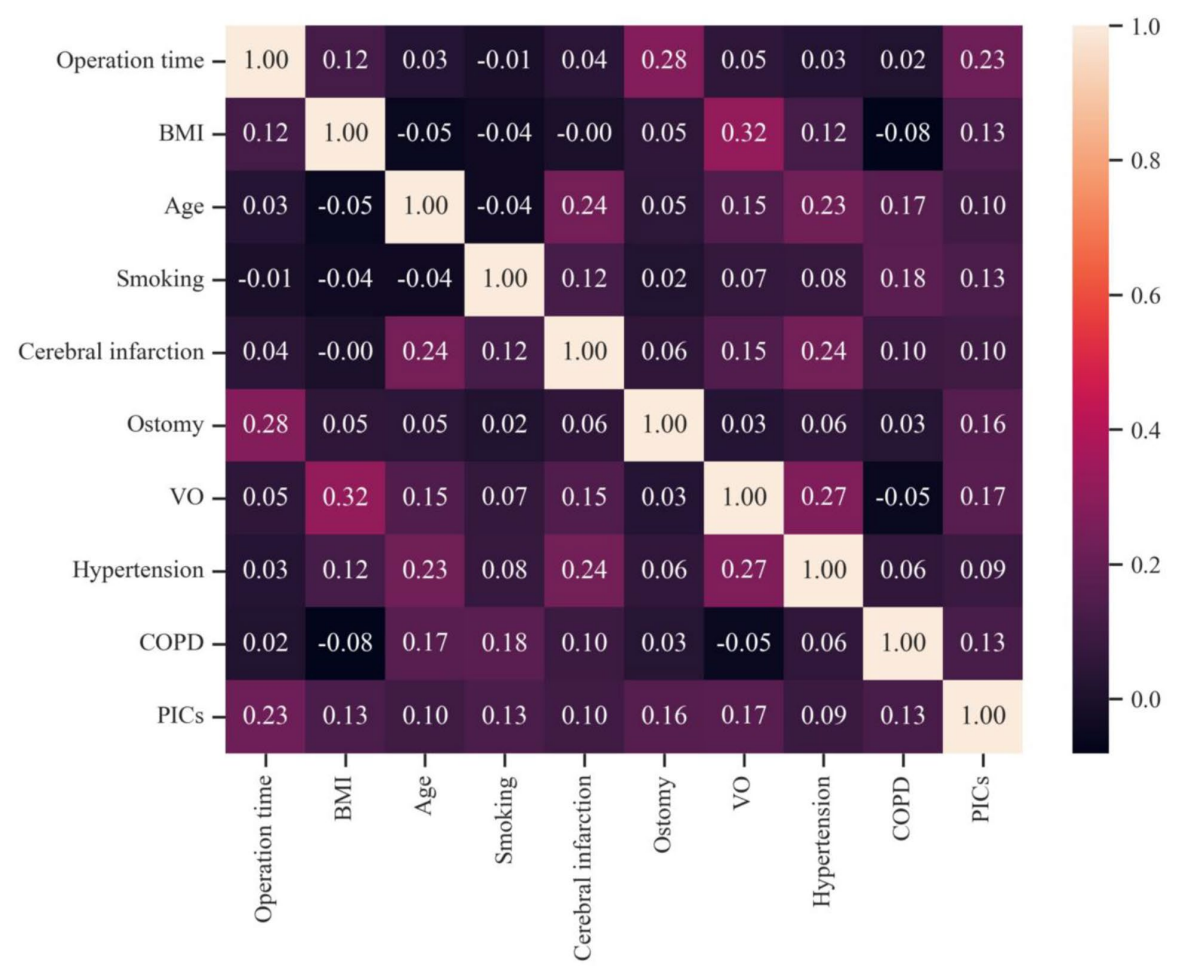
The Spearman correlation coefficient heatmap. The number in the box refers to the relationship between two variables. Correlation: weak, coefficient value < 0.5; strong, 0.5 ≤ coefficient value ≤ 0.7; stronger, coefficient value > 0.7. BMI, body mass index; VO, visceral obesity, visceral fat area ≥ 100 cm^2^; PICs, postoperative infectious complications

Table 3 displays the results of the univariate and multivariate analyses used to identify risk factors for postoperative infection-related complications. Univariate analysis showed that operation time ≥ 4 h, cerebral infarction, hypertension, high BMI, age ≥ 65 years, smoking, ostomy, VO, and COPD were associated with postoperative infection (*P* < 0.05). The Spearman correlation coefficient chart revealed no strong connection among these variables. (Figure. 3). Multivariate analysis showed that operation time ≥ 4 h (OR = 2.52, 95% CI 1.60 ~ 3.97, *P* < 0.001), smoking (OR = 2.04, 95% CI 1.16 ~ 3.59, *P* = 0.014), VO (OR = 2.01, 95% CI 1.17 ~ 3.48, *P* = 0.012), ostomy (OR = 1.65, 95% CI 1.04 ~ 2.61, *P* = 0.033), and COPD (OR = 2.23, 95% CI 1.09 ~ 4.57, *P* = 0.029) were independent risk factors for postoperative infectious complications.

**Table 3:** Univariate and multivariate analyses of factors associated with postoperative infectious complications

Feature	Univariable analysis			Multivariate analysis		
	*P* value	OR	95% CI	*P* value	OR	95% CI
Age	0.018^*^	1.64	1.09 ~ 2.48	0.159	1.41	0.88 ~ 2.26
Gender	0.336	0.81	0.54 ~ 1.24			
BMI	0.003^*^	1.90	1.24 ~ 2.90	0.071	1.56	0.96 ~ 2.52
SFA(cm^2^)	0.184	1.00	0.99 ~ 1.01			
VO	0.000^*^	2.52	1.55 ~ 4.08	0.012^*^	2.01	1.17 ~ 3.48
Smoking	0.003^*^	2.15	1.29 ~ 3.58	0.014^*^	2.04	1.16 ~ 3.59
Hypertension	0.049^*^	1.53	1.00 ~ 2.34	0.927	0.98	0.60 ~ 1.59
Diabetes	0.368	1.31	0.73 ~ 2.35			
COPD	0.005^*^	2.51	1.32 ~ 4.75	0.029^*^	2.23	1.09 ~ 4.57
Coronary heart disease	0.186	1.51	0.82 ~ 2.78			
Cerebral infarction	0.018^*^	1.90	1.12 ~ 3.25	0.418	1.28	0.70 ~ 2.34
Fatty liver	0.737	1.16	0.48 ~ 2.82			
Anemia	0.194	0.74	0.47 ~ 1.17			
Low albumin	0.782	0.94	0.61 ~ 1.45			
Fasting blood glucose	0.584	1.04	0.91 ~ 1.19			
WBC	0.255	0.94	0.84 ~ 1.05			
Neutrophil	0.106	0.90	0.79 ~ 1.02			
LMR	0.189	0.95	0.87 ~ 1.03			
Operation time	0.000^*^	2.95	1.94 ~ 4.49	0.000^*^	2.52	1.60 ~ 3.97
Surgical approach	0.281	1.48	0.72 ~ 3.03			
Perioperative blood infusion	0.207	1.42	0.82 ~ 2.45			
Ostomy	0.000^*^	2.19	1.44 ~ 3.33	0.033^*^	1.65	1.04 ~ 2.61
Pathology	0.981	1.01	0.57 ~ 1.78			
Tumor location	0.108	0.72	0.48 ~ 1.08			
Histologic type	0.248	1.35	0.81 ~ 2.25			
Tumor diameter (Max)	0.433	0.96	0.85 ~ 1.07			
Pathological stage	0.227	0.78	0.52 ~ 1.17			

Low albumin, albumin < 40 g/L; Anemia, hemoglobin concentration < 120 g/L for men and < 110 g/L for women

BMI, body mass index; VO, visceral obesity, visceral fat area ≥ 100 cm^2^; SFA, subcutaneous fat area; COPD, chronic obstructive pulmonary disease; LMR, lymphocyte to monocyte ratio; OR, odds ratio; CI, confidence interval

Age (65 years), BMI (25 kg/m^2^) and operation time (4 h) were set as binary variables

^*^Statistically significant (*P* < 0.05)

### Machine learning

The rate of postoperative infectious complications remained consistent in each train-test split. The AUCs for the six models are presented in the Additional file. 2. The LGBM model exhibited the largest AUC (0.74, 95% CI 0.68 ~ 0.81). We used the test set to perform internal validation, and the AUC was 0.67(95% CI 0.56 ~ 0.78).

We displayed the SHAP summary plot of LGBM (Additional file. 3). The SHAP summary plot of the LGBM model revealed that the factors that predict infection, in order of most to least significant, were operation time ≥ 4 h, VO, COPD, ostomy, and smoking.

## Discussion

Our present data showed that the relationship between VO and postoperative infection was pronounced. Although BMI and VO were both associated with infection in the univariate analysis, only VO remained an independent risk factor for infection in the multivariate analysis. Our research shed new light on the superior predictive value of VO compared with BMI for postoperative infectious complications.

Our study examined the relationship between fat parameters and BMI in participants. Our study showed a positive correlation between BMI and VFA. This was discovered between BMI and SFA as well. BMI, VFA, and SFA all represented the status of fat accumulation, which could explain the positive correlation between VFA and BMI, SFA and BMI. VFAs and SFAs are two major components of adipose tissue and were also positively correlated with each other in our study. SAT was the largest fat repository of people. When the SAT reached its maximum, extra energy was stored as VAT due to excessive energy intake or low energy consumption [21].

In this study, there were only 16 patients (3.1%) with BMI ≥ 30 kg/m^2^. Thus, it is difficult to evaluate the influence of BMI ≥ 30 kg/m^2^ on colorectal cancer resection in Chinese people. There are differences in obesity prevalence and body fat distribution among ethnic populations, and the Asian population tends to accumulate visceral fat [22]. Therefore, we adopted the Asian-Pacific standard to define obesity. In our study, high BMI was not associated with diabetes, coronary heart disease, or cerebral infarction compared with low BMI. In contrast, preoperative comorbidities were all connected with VO, except COPD. These results confirmed past reports on the correlation between VO and metabolic syndrome [23].

Our study suggested that VO and high BMI were associated with total postoperative infection. To our knowledge, domestic and international studies on the relationship between VO and postoperative infection primarily focus on a single infection (e.g., wound infection and anastomotic leakage), and few studies have comprehensively analyzed the correlation between VO and total postoperative infection-related complications for colorectal cancer patients. In our analysis of the single type of infectious complication, VO was not associated with anastomotic fistula and urinary infection, which might be due to their low incidence in our study. BMI was hardly related to any kind of infection. Moreover, we found that VO and high BMI were unconnected with postoperative hospital stays and the rate of transfer to the intensive care unit after surgery, which was not consistent with previous conclusions. This might be influenced by the postoperative judgment of surgeons for patients or the severity of infectious complications.

According to the literature, VO is associated with chronic inflammatory responses and insulin resistance [24]. On the one hand, insulin resistance could slow the healing process of the wound, and inflammatory reactions might produce inflammatory factors that influence homeostasis, such as CRP and IL-6 [25]. Patients with a VO-associated chronic inflammatory state might react to different immunological responses to surgery. This idea requires further investigation since it might help to identify new perioperative strategies for preventing postoperative infection. On the other hand, patients with VO had excessive fat accumulation, and surgeons exposed the surgical field of vision difficulty, which increased the surgical difficulties and prolonged the operation time. Moreover, patients with VO had high blood lipid levels and microcirculation disturbance [26], which resulted in poor oxygen supply and prevented tissue repair. In summary, anatomical factors, chronic inflammatory response, insulin resistance, and microcirculation disturbance jointly contributed to infectious outcomes following colorectal cancer resection.

In this study, operation time ≥ 4 h, ostomy, smoking, and COPD were also independent risk factors for postoperative infection. The relationship between operation time and infection has been previously reported [27]. Our study found that when the operation time was longer than 4 h, the risk of infection in patients increased 2.52-fold. With the extension of operation time, many bacteria invaded the surgical area, which decreased the ability to fight infection for patients. On the other hand, patients consumed more anesthetics, which could inhibit the cough reflex and increase the risk of respiratory infections. Ramzi Amri [28] analyzed the baseline factors of postoperative infection and demonstrated that lifestyle factors, such as smoking, sharply increased infectious risk. Our study also found the same result. Smokers are more susceptible to respiratory tract infections following surgery. Smoking induces increased permeability of epithelial cells, mucus overproduction, and impaired mucociliary clearance. This results in an increased release of proinflammatory cytokines and chemokines, aggravating inflammation and promoting postoperative infection [29]. Similarly, lung function was impaired in patients with COPD, which made it easy to induce postoperative respiratory system infections [30]. Because the wound was close to the ostomy in patients with postoperative ostomy, the risk of infection also increased [31]. Additionally, some recognized variables were not included as independent risk factors in our study, which included diabetes [32] and surgery approach [33]. This might be connected with the population of our research center and surgical techniques.

The characteristic of the study was the use of SHAP values to display the results of machine learning methods, which helped models to be visualized and easily understood. We trained and validated machine learning models using five risk factors in this retrospective cohort study. According to the SHAP summary plot of the LGBM model, the effect of VO on postoperative infection was second only to operation time. Currently, with the development of software technology, it is more accessible to measure VFA. Thus, VFA could be recommended as a routine preoperative test for colorectal cancer to assess VO, which helped to predict the risk profile for infection in colorectal cancer patients.

### Study strengths and limitations

One of the major strengths of our study lies in the utilization of VO as a criterion for obesity assessment to evaluate the distribution of fat tissue. Furthermore, we developed predictive models utilizing six machine-learning approaches and visualized the optimal model using the SHAP value. This study had several limitations. First, our study included relatively few patients, and the data came from a single center. The results of machine learning approaches might vary for different distributions of patient characteristics or different institutions from which data came. The model we trained might be applied to the Asian-Pacific population at best. Second, it is unclear whether the established risk models can be translated into actual clinical benefits for patients in clinical practice. Therefore, many prospective, multicenter studies are needed for further validation and evaluation.

## Conclusions

In this study, CT-measured VO was found to be more sensitive to metabolic comorbidities and postoperative infection than BMI. Therefore, we recommended VFA as a preoperative test to assess VO for colorectal cancer patients. We established the LGBM predictive model using machine learning methods that could predict the individual risk of postoperative infection for colorectal cancer patients.

### Electronic supplementary material

Below is the link to the electronic supplementary material.

Supplementary Material 1

## Data Availability

The datasets used and/or analyzed during the current study are available from the corresponding author upon reasonable request.

## List of abbreviations

AUC: Area under the ROC curve
BMI: Body mass index
CI: Confidence interval
COPD: Chronic obstructive pulmonary disease
CRC: Colorectal cancer
CRP: C-reactive protein
CT: Computed tomography
DT: Decision tree
GBDT: Gradient boosting decision tree
GridSearchCV: Cross validated gridsearch
Hb: Hemoglobin
HU: Hounsfield unit
LGBM: Light gradient boosting machine
LMR: Lymphocyte‑to‑monocyte ratio
LR: Logistic regression
OR: Odds ratio
PICs: Postoperative infective complications
RF: Random forest
ROC: Receiver operating characteristic
SFA: Subcutaneous fat area
SHAP: Shapley Additive explanation
SSI: Surgical site infection
VAT: Visceral adipose tissue
VFA: Visceral fat area
VO: Visceral obesity
WHO: World health organization
XGBoost: Extreme Gradient Boosting
