# Supplementary material for: Impact of visceral obesity on infectious complications after resection for colorectal cancer: a retrospective cohort study
Source: Lipids Health Dis. 2023 Aug 31;22:139. doi: 10.1186/s12944-023-01890-4 (PMC10469994; doi:10.1186/s12944-023-01890-4)

Additional file. 1. Flow diagram for the study selection process

Additional file. 2. Comparison of AUCs among the six machine learning models. LR, logistic regression; RF, random forest; DT, decision tree; LGBM, light gradient boosting machine; XGBoost, extreme gradient boosting; GBDT, gradient boosting decision tree.

Additional file. 3. SHAP summary plot of the LGBM model. The higher the SHAP value of a feature, the higher the probability of postoperative infective complications. Each dot represents a sample. Red represents higher feature values, and blue represents lower feature values. VO, visceral obesity, visceral fat area ≥ 100 cm^2^; COPD, chronic obstructive pulmonary disease; SHAP, SHAPley Additive exPlanations; LGBM, light gradient boosting machine.

Additional file. 1


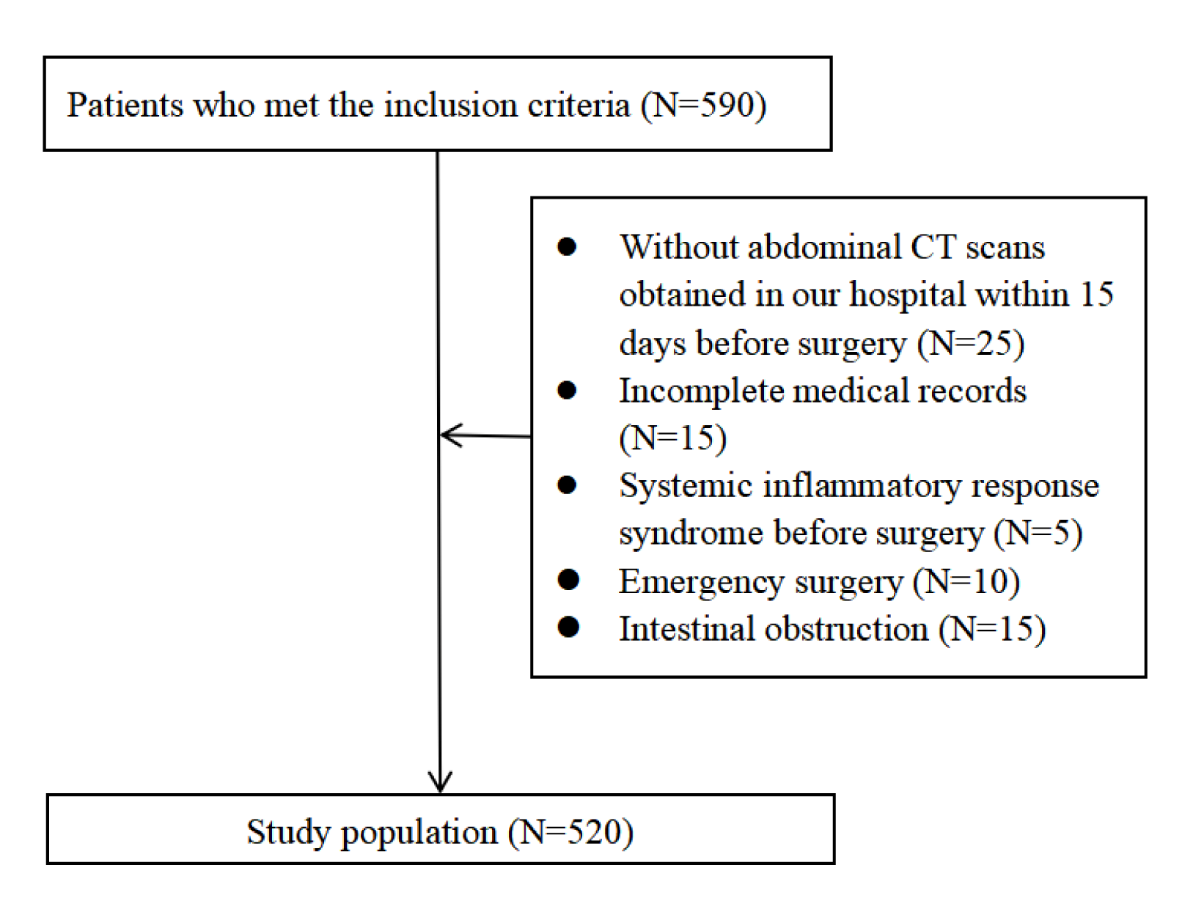


Additional file. 2


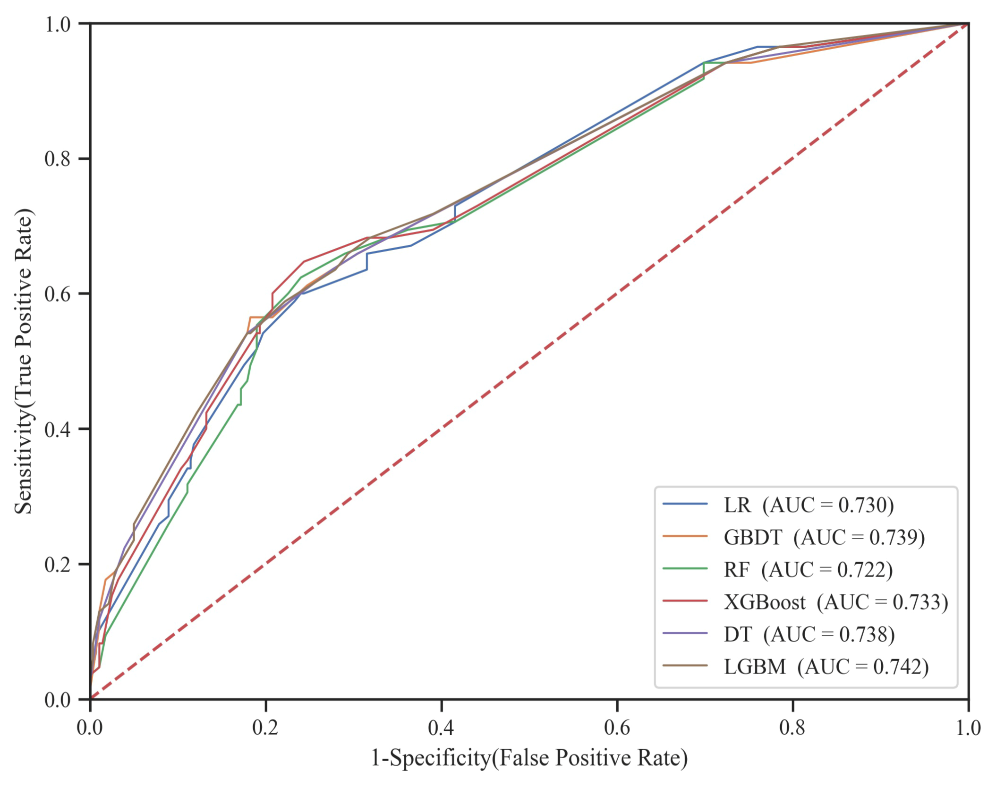


| Model | AUC (95% CI) |
| --- | --- |
| LR | 0.730 (95% CI 0.664 ~ 0.796) |
| GBDT | 0.739 (95% CI 0.673 ~ 0.804) |
| RF | 0.722 (95% CI 0.655 ~ 0.788) |
| XGBoost | 0.733 (95% CI 0.667 ~ 0.798) |
| DT | 0.738 (95% CI 0.673 ~ 0.803) |
| LGBM | 0.742 (95% CI 0.676 ~ 0.807) |

Additional file. 3


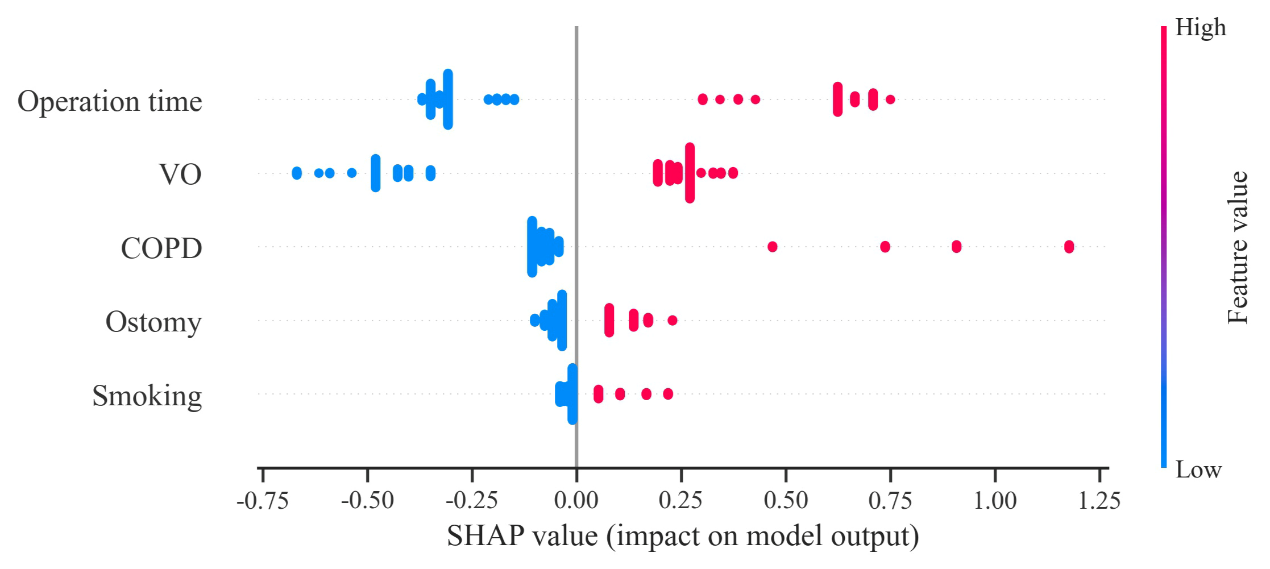

Supplement: Supplementary file 1 — Supplementary Material 1 [file 12944_2023_1890_MOESM1_ESM.docx]
